# Autologous Mesenchymal Stem Cells Show More Benefit on Systolic Function Compared to Bone Marrow Mononuclear Cells in a Porcine Model of Chronic Myocardial Infarction

**DOI:** 10.1007/s12265-015-9643-3

**Published:** 2015-09-17

**Authors:** T. I. G. van der Spoel, W. A. Gathier, S. Koudstaal, F. van Slochteren, S. Jansen of Lorkeers, J. P. G. Sluijter, I. E. Hoefer, P. Steendijk, M. J. M. Cramer, P. A. Doevendans, E. van Belle, S. A. J. Chamuleau

**Affiliations:** Department of Cardiology, Division Heart and Lungs, University Medical Center Utrecht, Rm E03.511, Heidelberglaan 100, 3584 CX Utrecht, The Netherlands; Interuniversity Cardiology Institute of the Netherlands (ICIN), Utrecht, The Netherlands; Department of Experimental Cardiology, Utrecht, The Netherlands; Department of Cardiology, Leiden University Medical Center, Leiden, The Netherlands

**Keywords:** Stem cells, Ischemic cardiomyopathy, Mesenchymal stem cell, Systolic function

## Abstract

Cardiac cell therapy is a strategy to treat patients with chronic myocardial infarction (MI). No consensus exists regarding the optimal cell type. First, a comparison between autologous bone marrow-derived mononuclear cells (BMMNC) and mesenchymal stem cells (MSC) on therapeutic efficacy after MI was performed. Next, the effect of repetitive, NOGA-guided transendocardial injection was determined via a crossover design. Nineteen pigs were allocated in three groups: (1) placebo (at 4 and 8 weeks), (2) MSC (followed by placebo at 8 weeks), or (3) BMMNC (followed by MSC at 8 weeks) delivery including a priming strategy to enhance MSC effect. At 4 weeks, ejection fraction (EF) was significantly improved after MSC injection and not by BMMNC injection. After 8 weeks, no difference was observed in EF between cell-treated groups demonstrating the positive systolic effect of MSC. This study showed that MSC rather than BMMNC injection improves systolic function in chronic MI.

## Introduction

Ischemic heart failure remains a major cause of morbidity and mortality [1]. Stem cell therapy emerged as an innovative and attractive therapeutic approach for patients with chronic myocardial infarction (MI). The ultimate goal of this treatment is to support and enhance the endogenous repair mechanisms by replacing dysfunctional cardiomyocytes and inducing angiogenesis.

In clinical and preclinical studies, a modest improvement in left ventricular ejection fraction (LVEF) was observed using a single injection of bone marrow cells after MI [2, 3]. Our preclinical meta-analysis showed that the choice of cell type is an important significant predictor of improvement in LVEF [3] suggesting a trend towards more pronounced effects of mesenchymal stem cells (MSC). Till now, bone marrow mononuclear cells (BMMNC) and MSC have been well studied in patients with ischemic heart disease [4]. However, it is known that functional differences between MSC and BMMNC exist [3]. A direct comparison on functional endpoints between these cell types has not been performed so far. We hypothesized that pretreatment of the area of interest could be helpful to further enhance the effects of MSC. Thus, we incorporated a repetitive cell injection strategy in the study design.

Percutaneous transendocardial (TE) delivery, guided by electromechanical mapping (NOGA), was shown to be safe in patients with chronic ischemic cardiomyopathy [5] and has the advantage of detecting hibernating myocardium which is the area that will probably profit most from cell delivery [6].

Our objective was to determine the most potent regenerative strategy using autologous bone marrow cell types, i.e., BMMNC and MSC, in a large animal model of ischemia/reperfusion injury. First, a direct comparison between BMMNC and MSC was performed 4 weeks after transplantation. Second, the effect of repetitive injection after initial priming was determined by including a crossover with MSC in the BMMNC group with an additional follow-up period of 4 weeks.

## Method

### Animals

Nineteen female Dutch Landrace pigs received humane care in compliance with the “Guide for the Care and Use of Laboratory Animals,” published by the National Institutes of Health (National Institutes of Health publication 85-23, revised 1985). The study protocol was approved by the Animal Experimentation Committee of the University of Utrecht.

### Study Design

**Fig. 1:**
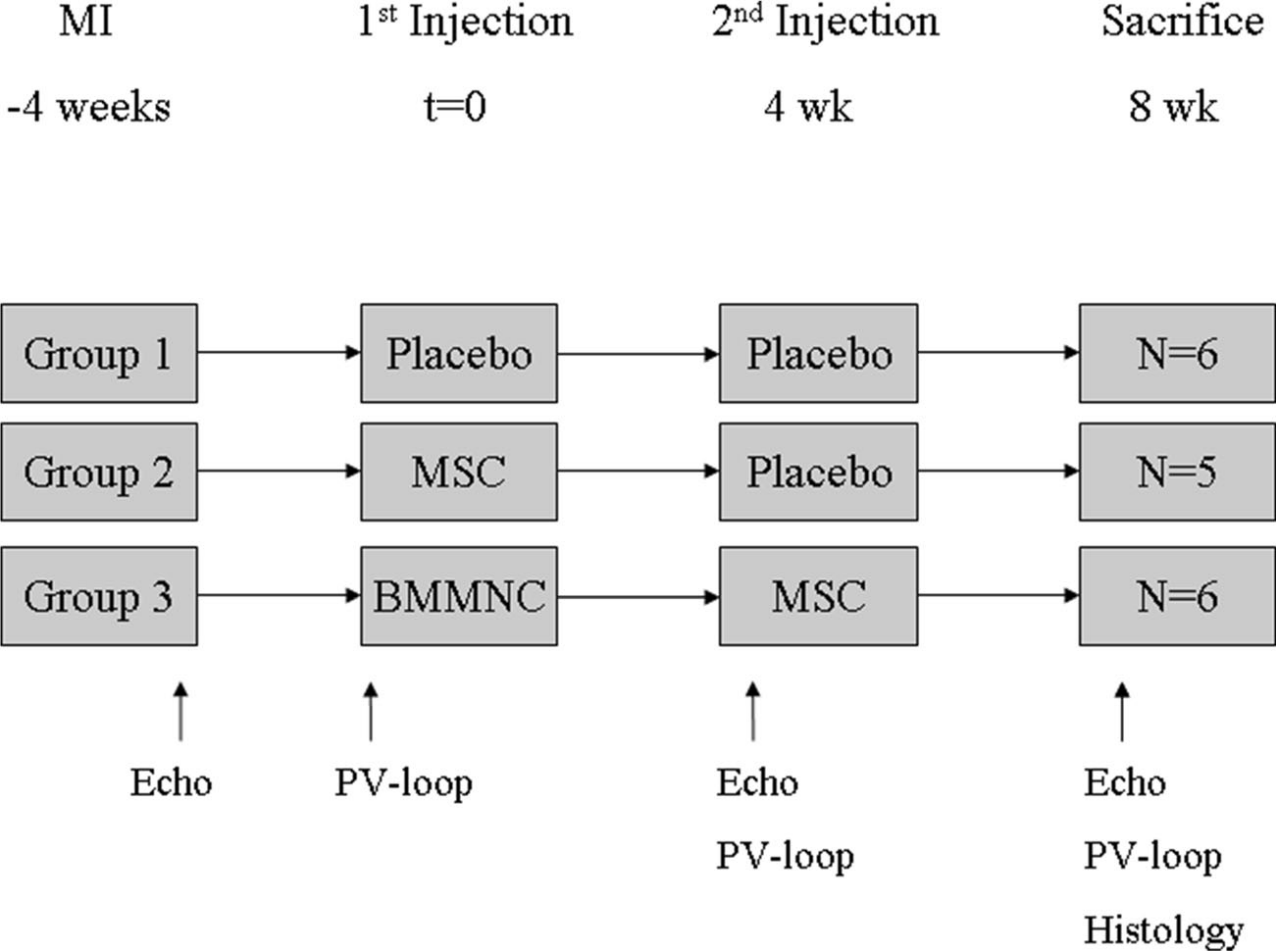
Study design. *BMMNC* bone marrow mononuclear cells, *Echo* echocardiography, *MI* myocardial infarction, *MSC* mesenchymal stem cells, *PV loop* pressure-volume loop

Animals were allocated to one of three groups: (group 1) placebo (phosphate buffered saline (PBS), Invitrogen, Carlsbad, CA, USA), (group 2) 10^7^ autologous MSC, or (group 3) 10^7^ autologous BMMNC injection at 4 weeks. Eight weeks after MI (thus, 4 weeks after initial injection), the animals in group 3 received an additional injection of MSC to determine whether priming could rescue the damaged myocardium, while the other groups received an injection with PBS for control purposes. Twelve weeks after the initial MI, the animals were euthanized and tissue was prepared for histology. Cardiac function was assessed by pressure-volume (PV) loops and echocardiography. The study design is shown in Fig. 1.

### Premedication and Anesthesia

After an overnight fast, animals were sedated with an intramuscular injection of ketamin (10 mg/kg), midazolam (0.5 mg/kg), and atropin (0.04 mg/kg). Next, thiopental (4 mg/kg) was administered intravenously before intubation. They were intubated with an endotracheal tube and anesthetized in the supine position. The animals were mechanically ventilated with the use of a positive pressure ventilator with a mix of oxygen and air (FiO2 0.5). General anesthesia/analgesia was maintained with midazolam (0.5 mg/kg/h, Roche, Woerden, the Netherlands), sufentanyl citrate (2 μg/kg/h, Janssen-Cilag, Tilburg, the Netherlands), and pancuronium bromid (0.1 mg/kg/h, Organon, Oss, the Netherlands). Metoprolol (Centrafarm, Etten-Leur, the Netherlands) was administered intravenously (5 mg) to reduce the mechanical irritation of the heart. During surgery, animals were anticoagulated with heparin (ACT > 250 s). At the end of the experiment, the animals were euthanized by pentobarbital overdose.

### Myocardial Ischemia/Reperfusion Model

During the entire procedure, electrocardiogram, arterial pressure, and capnogram were continuously monitored. Prior to MI, all animals received an oral dose of amiodarone (400 mg/day; starting 10 days prior to MI) and clopidogrel (75 mg/day; starting 3 days prior to MI; Sanofi Aventis, Gouda, the Netherlands) [7]. A bolus of 500 mg acetylic salicylic acid (Centrafarm, Etten-Leur, the Netherlands) was given the day before the occlusion. Myocardial infarction was created by a percutaneous balloon of equivalent size to the proximal left circumflex artery (LCX). The balloon was inflated for 75 min at 5–8 atm [8]. Complete occlusion of the LCX was confirmed by angiography. To prevent ventricular arrhythmias, 300 mg amiodarone (Centrafarm, Etten-Leur, the Netherlands) intravenously was given. External defibrillation (150–200 J) was used when ventricular fibrillation occurred. After the procedure, coronary angiography was performed to confirm vessel patency. After recovery, the animals received daily an oral dose of 50 mg metoprolol, 400 mg amiodarone, 75 mg clopidogrel, and 160 mg acetylic salicylic acid until termination to prevent thrombosis and arrhythmias [7].

### MSC Culture and Labeling

Bone marrow was aspirated (35–40 mL) from the sternum by a heparinized syringe. BMMNC were isolated by Ficoll density gradient centrifugation and frozen in 10 % DMSO and 90 % culture medium.

MSC were isolated and characterized as previously described [9]. Autologous MSC were cultured at 37 °C in Alpha MEM (Invitrogen, Carlsbad, CA, USA), supplemented with 10 % FBS, heparin, and 1 % penicillin/streptomyocin. Cells were cultured, replacing medium every 3 days and used between passage 5 and 7. Before injection, cells were resuspended in 2 mL PBS and viability was assessed via trypan-blue (Sigma-Aldrich, St. Louis, MO, USA) counting.

### Transendocardial Delivery

To enable TE injection, an 8-F sheath was placed in a carotid artery. Next, a mapping catheter (Biosense Webster, Cordis, Johnson & Johnson, USA) was placed retrogradely through the aortic valve into the left ventricle (LV). First, a 3-dimensional electromechanical map of the LV was obtained using the NOGA system (Biosense Webster, Cordis, Johnson & Johnson, USA), as described before [10, 11]. Hereafter, 10 injections of 0.2 mL were slowly placed using the MYOSTAR® injection catheter (Biosense Webster, Cordis, Johnson & Johnson, Diamond Bar, USA). Two injections were placed in the infarct zone and eight in the border zone. Four weeks after the first injection, this procedure was repeated and the second injections were given at the same location. Injections were only given in areas with a unipolar voltage greater than 6 mV [10, 11].

### Echocardiography

A transthoracic echocardiogram (5-MHz probe, IE-33, Philips, Best, the Netherlands) was performed directly after MI, 8 weeks after MI, and at sacrifice as described before [7]. Short axis images were obtained at the papillary level, and three consecutive cardiac cycles were acquired. Wall thickness (WT) of the posterolateral wall was assessed in end-systole and end-diastole. The left ventricular internal area (LVIA) was obtained without including the papillary muscles in end-systole and end-diastole. The fractional area shortening was calculated as ((LVIAendiastole − LVIAendsystole)/LVIAenddiastole) × 100.

### Pressure-Volume Loop protocol

Pressure-volume loops were obtained using a 7-F conductance catheter that was inserted via a carotid artery and placed along the long axis of the LV. The catheter was connected with a signal processor (Leycom CFL, CD Leycom, Zoetermeer, the Netherlands). The correct position of the conductance catheter was verified by angiography and by inspection of the segmental conductance signals. The conductance signals were calibrated by thermodilution and hypertonic saline dilution via a 7-F Swan-Ganz catheter that was placed into the right or left pulmonary artery [12, 13]. Data were collected during steady-state conditions with the respirator systems turned off at end-expiration. From these signals, hemodynamic indices were derived. Data analysis and calculations were performed using custom-made software (CD Leycom, Zoetermeer, the Netherlands), as previously described [14]. Parameters of global systolic and diastolic function were calculated during steady-state conditions at 4, 8, and 12 weeks after MI. Cardiac output (CO) measured by Swan-Ganz was corrected by multiplying each measurement with 0.62. This number was based on the following equation (CO Swan-Ganz at sacrifice/CO transonic aorta flow probe at sacrifice). The isovolumic relaxation time constant (Tau) was calculated by phase-plot analysis. The end-systolic pressure-volume relationship was measured by its slope end-systolic elastance (Ees). Diastolic stiffness (Eed) was determined as the lineair slope of the end-diastolic pressure-volume relationship. Both were calculated by single-beat analysis as described earlier [15].

### Histology

After euthanasia, the LV was weighed and tissue samples from the infarct, borderzone, and remote region of the heart were obtained. Samples were fixed in 4 % formalin at room temperature. Before cutting 5-μm sections, samples were embedded in paraffin for analysis. For quantification of collagen content, picrosirius red staining and detection with circularly polarized light and digital image microscopy was used [16]. Five random images at ×20 magnification of the infarcted, borderzone, and remote area were obtained per animal. After conversion into gray value images, the average number of grey values was expressed as a mean grey value per square micrometer. Capillary density was assessed by Lectin staining (Sigma-Aldrich) and counterstained with Hematoxilin and Eosin to identify nuclei. In total, five fields per section at ×20 magnification were counted per animal per zone.

### Statistical Analysis

Values derived from echocardiography were analyzed in a blinded fashion. For statistical analysis, we used a linear mixed effects model to account for repeated measurements on each animal. In this model, we included a generalized estimation equations-type matrix to account for the association between residual covariance, e.g., time point of measurement (8 and 12 weeks after MI). Statistical comparison of data between groups was done using a one-way ANOVA with a post hoc Tukey or Kruskal-Wallis test. Data are presented as mean ± SE or median with interquartile ranges in case of non-normal distributed data. All statistical analyses were performed using SPSS 18.1.1, and *P* values <0.05 were considered statistically significant.

## Results

### Procedural Data

In total, 19 animals underwent the MI procedure. One animal in the placebo died due to severe heart failure evidenced by obduction (group 1; day 71 after MI), and one animal had to be terminated for reaching a human-defined endpoint due to an abscess at the right foot not related to the study (group 2). MSC viability (group 2, 92 ± 4 % vs. group 3, 93 ± 1 %; *P* = 0.10) and number of MSC (group 2, 1.0 ± 0.1 × 10^7^ vs. group 3, 0.9 ± 0.2 × 10^7^; *P* = 0.10) did not differ between the cell-treated groups. BMMNC viability was 92 ± 4 % and the injected number 1.7 ± 0.2 × 10^7^. No cardiac tamponade or sustained ventricular arrhythmias were observed after any cell or placebo injection.

### Comparison Between MSC and BMMNC on Cardiac Function at 4 weeks after Cell Transplantation

**Fig. 2:**
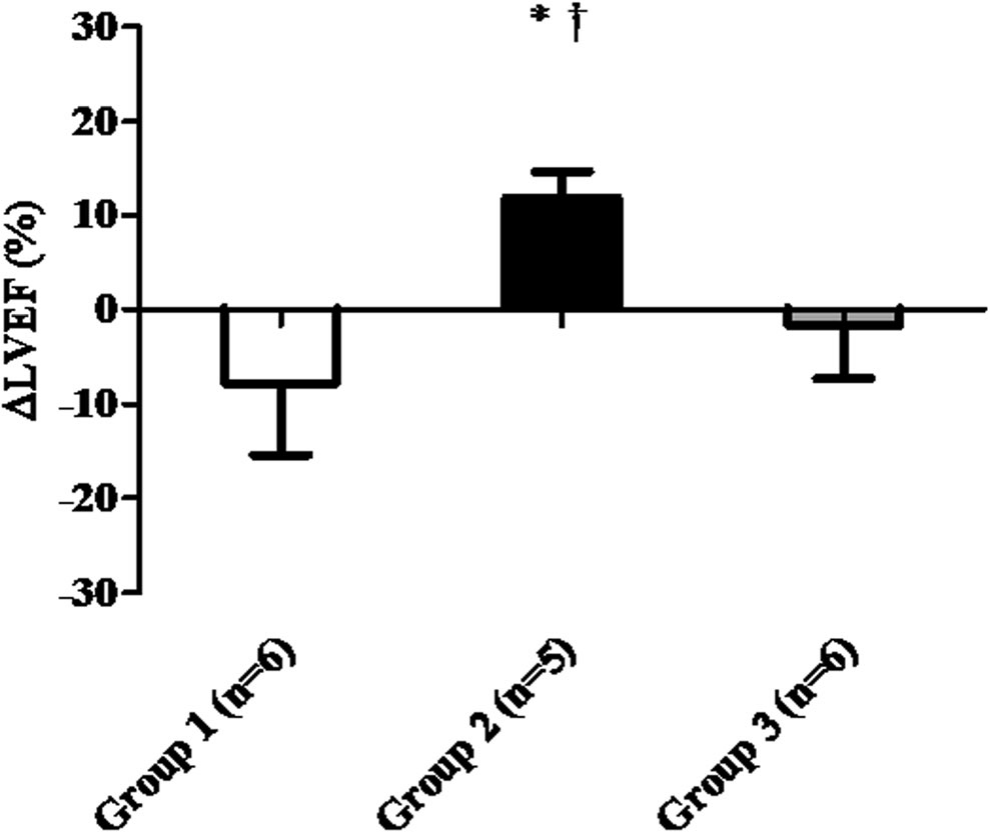
Effects at 4 weeks after cell therapy: group 2 (MSC injection) improves systolic function compared to group 3 (BMMNC) and group 1 (placebo). Percentage of change in LVEF between baseline and 4 weeks after injection in each treatment group. *P < 0.01 compared to group 1. †*P* = 0.028 compared to group 3. *LVEF* left ventricular ejection fraction§

Four weeks after MI (baseline), no difference in LVEF between groups was observed (*P* = 0.30; Table 1). When comparing LVEF differences between baseline and 4 weeks after injection (Fig. 2), placebo-treated animals showed a reduction in LVEF whereas in MSC-treated animals, LVEF was significantly improved (group 2, 11.9 ± 3 % vs. group 1, −7.8 ± 8 %; *P* = 0.002). Animals treated with MSC showed a tendency for having a decrease in ∆ESV (group 2, −6.0 ± 7 mL vs. group 1, 10 ± 10 mL; *P* = 0.10). No significant difference in ∆LVEF between BMMNC and placebo treatment was observed (group 3, −1.6 ± 6 % vs. group 1, −7.8 ± 8 %; *P* = 0.748). Consequently, MSC injection led to a significant increase in ∆LVEF compared to BMMNC injection (group 2, 11.9 ± 3 % vs. group 3, −1.6 ± 6 %; *P* = 0.028) but also significantly improved ∆CO (group 2, 0.7 ± 0.3 L/min vs. group 3, −0.4 ± 0.4 L/min; *P* = 0.037) and thereby reflects an increased systolic cardiac performance. After BMMNC injection, a trend for impaired ∆dP/dt_MAX_ was observed compared to MSC-treated animals (group 2, −38 ± 154 mmHg vs. group 3, −277 ± 57 mmHg; *P* = 0.08).

**Table 1:** Hemodynamics derived from pressure-volume loops at baseline, before the second injection and at sacrifice

Hemodynamics	Baseline (4 weeks after MI)			Injection (8 weeks after MI)			Sacrifice (12 weeks after MI)		
Parameter	Group 1	Group 2	Group 3	Group 1	Group 2	Group 3	Group 1	Group 2	Group 3
General									
Weight (kg)	73 ± 3	71 ± 2	73 ± 1	80 ± 3	76 ± 2	76 ± 1	83 ± 3	82 ± 3	82 ± 1
LV weight (g)							168 ± 7	159 ± 5	175 ± 9
FAS (%)				50 ± 5	55 ± 3	47 ± 2	50 ± 2	51 ± 2	43 ± 3
HR (beats/min)	52 ± 2	59 ± 4	52 ± 6	63 ± 8	56 ± 1	57 ± 7	51 ± 7	55 ± 1	53 ± 4
CO (L/min)	3.5 ± 0.3	2.8 ± 0.2	3.0 ± 0.4	3.2 ± 0.2	3.5 ± 0.3^§^	2.7 ± 0.2	2.8 ± 0.5	3.5 ± 0.3	3.1 ± 0.1
Systole									
ESV (mL)	41 ± 3	37 ± 4	49 ± 7	50 ± 12	31 ± 8	44 ± 7	51 ± 11	23 ± 5^#^	30 ± 6*
ESP (mmHg)	96 ± 7	87 ± 11	100 ± 5	86 ± 7	90 ± 8	85 ± 4	91 ± 7	81 ± 5	74 ± 4*
EF (%)	62 ± 2	57 ± 2	55 ± 5	54 ± 7	69 ± 3^#§^	54 ± 5	52 ± 3	74 ± 3^#^	69 ± 4*
dP/dt_MAX_ (mmHg/s)	1586 ± 131	1390 ± 208	1374 ± 46	1372 ± 152	1351 ± 134	1096 ± 64	1460 ± 102	1402 ± 40^§^	1033 ± 71*
Ees (mmHg/ml)	3.9 ± 0.5	4.2 ± 0.6	3.7 ± 0.1	3.7 ± 0.7	3.7 ± 0.4	3.2 ± 0.2	4.1 ± 0.9	3.7 ± 0.5	2.5 ± 0.3*
Diastole									
EDV (mL)	107 ± 8	85 ± 5	109 ± 6	106 ± 13	92 ± 13	95 ± 9	106 ± 16	86 ± 9	91 ± 10
EDP (mmHg)	16 ± 1	13 ± 1	16 ± 1	14 ± 2	16 ± 1	13 ± 2	15 ± 1	14 ± 2	11 ± 1*^§^
dP/dt_MIN_ (mmHg/s)	−1428 ± 131	−1345 ± 165	−1393 ± 91	−1350 ± 224	−1447 ± 119	−1275 ± 99	−1239 ± 224	−1328 ± 99	−1148 ± 93
PHT (ms)	34 ± 2	31 ± 2	39 ± 3	36 ± 7	31 ± 1	34 ± 3	44 ± 7	28 ± 1^#^	31 ± 1*
Tau (ms)	58 ± 4	52 ± 4	67 ± 6	62 ± 14	51 ± 2	57 ± 5	72 ± 16	48 ± 2	49 ± 2*
Eed (mmHg/mL)	0.38 ± 0.04	0.42 ± 0.02	0.37 ± 0.06	0.46 ± 0.8*	0.54 ± 0.06^§^	0.29 ± 0.02	0.30 ± 0.04	0.38 ± 0.07	0.24 ± 0.03

Data are presented as mean ± SE

*CO* cardiac output, *HR* heart rate, *EDP* end-diastolic pressure, *ESP* end-systolic pressure, *dP/dt*_*MAX*_ maximal rate of LV pressure increase, *dP/dt*_*MIN*_ minimal rate of LV pressure decrease, *EDV* end-diastolic volume, *Eed* myocardial stiffness, *Ees* end-systolic elastance, *ESV* end-systolic volume, *EF* ejection fraction, *FAS* fractional area shortening, *LV* left ventricle, *PHT* pressure halftime, *Tau* isovolumic relaxation time constant

*P < 0.05 BMMNC vs. placebo, ^#^ *P* < 0.01 MSC vs. placebo, ^§^ *P* < 0.05 MSC vs. BMMNC

Regarding global diastolic function, no significant difference in ∆end-diastolic volume between groups could be observed (group 1, −0.2 ± 4 mL; group 2, 7.7 ± 13 mL; group 3, −14 ± 8 mL; all *P* > 0.1). In addition, dP/dt_MIN_, Tau, end-diastolic pressure (EDP), and pressure halftime (PHT) were similar in the different treatment groups (Table 1). However, passive diastolic function was improved in the BMMNC group compared to the other groups, indicated by ∆Eed (group 1, 0.08 ± 0.05 mmHg/mL; group 2, 0.12 ± 0.08 mmHg/mL; group 3, −0.08 ± 0.05 mmHg/mL; BMMNC vs. placebo, *P* = 0.04; MSC vs. BMMNC, *P* = 0.004; MSC vs. placebo, *P* = 0.349).

Directly after MI, echocardiographic recordings showed that end-systolic WT was similar between groups (group 1, 1.25 ± 0.2 cm; group 2, 1.38 ± 0.1 cm; group 3, 1.01 ± 0.2 cm; *P* = 0.78). Also, no difference in end-diastolic WT was observed (group 1, 1.21 ± 0.3 cm; group 2, 1.18 ± 0.3 cm; group 3, 1.01 ± 0.2 cm; *P* = 0.48). Four weeks after treatment, no significant effect on ∆end-diastolic WT (group 1, 0.03 ± 0.06 cm; group 2, 0.01 ± 0.03 cm; group 3 0.10 ± 0.05 cm) and ∆end-systolic WT (group 1, 0.28 ± 0.07 cm; group 2, 0.06 ± 0.06 cm; group 3, 0.16 ± 0.09 cm) was found.

### Effect of Repeated Cell Injection on Cardiac Function at 8 weeks after Cell Transplantation

Since no effect of BMMNC on ∆LVEF was observed, we now did not expect a synergistic effect of repetitive BMMNC injection in group 3. However, we now were able to study whether a second injection of MSC could rescue the damaged myocardium.

**Fig. 3:**
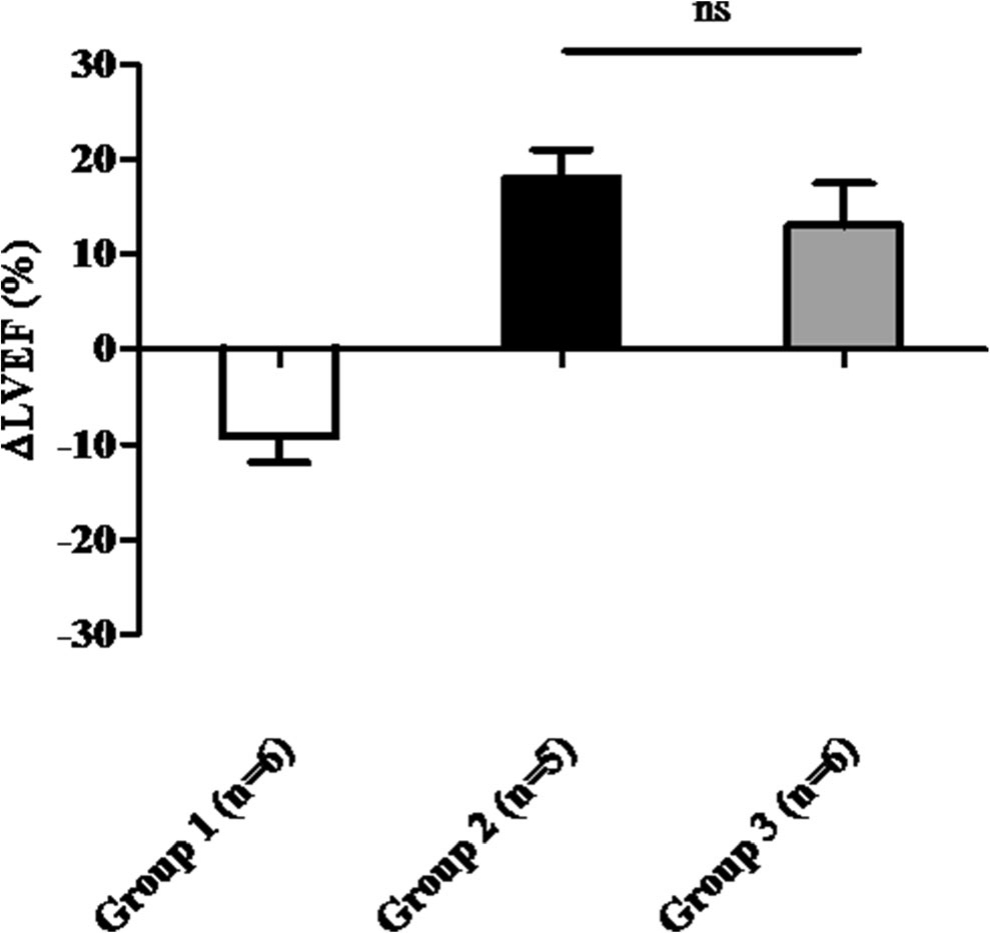
Effect at 8 weeks after (repeated) cell therapy: no difference on EF between single (group 2) versus pretreated injections (group 3) with MSC. No significant effect on ∆LVEF (baseline and 8 weeks after injection) between single and repeated cell injection was observed. *LVEF* left ventricular ejection fraction

When comparing ∆LVEF between baseline and at sacrifice (Fig. 3), placebo-treated animals showed a reduction in ∆LVEF, whereas in cell-treated animals, ∆EF was significantly improved (group 2, 18 ± 3 %; group 3, 13 ± 4 % vs. group 1, −9 ± 3 %; all *P* < 0.01) caused by a significant reduction in ∆ESV (group 2, −14 ± 4 mL; group 3, −20 ± 4 mL vs. group 1, 11 ± 10 mL; all *P* < 0.01). However, no difference in ∆EF or ∆ESV between single MSC injection and repeated cell delivery could be observed (*P* = 0.28 and *P* = 0.79). Contractility measured by ∆dP/dt_MAX_ was significantly increased after single MSC injection, compared to BMMNC and MSC injection (group 2, 105 ± 193 mmHg; group 3, −340 ± 63 mL; *P* = 0.003). In fact, the second MSC injection on top of the first BMMNC injection (without significant difference compared to placebo) once more revealed the magnitude of effect on systolic function by MSC.

Overall, both cell groups showed an improvement in diastolic active relaxation parameters compared to placebo-treated animals. This was reflected by a shortened ∆Tau and decreased ∆PHT. Myocardial stiffness (Eed) was unaffected by cell therapy. No statistical difference in active and passive diastolic function between the cell-treated groups could be observed, except for EDP.

No significant difference in echocardiographic parameters between single cell injection and repeated cell injection was observed.

### Capillary Density and Collagen

Histological samples were not available for two animals (both group 1). Due to technical issues, in 175 of the 225 samples (78 %), representative Lectin stainings were obtained and used for analysis. Both collagen and vascular density data showed a non-normal distribution.

**Fig. 4:**
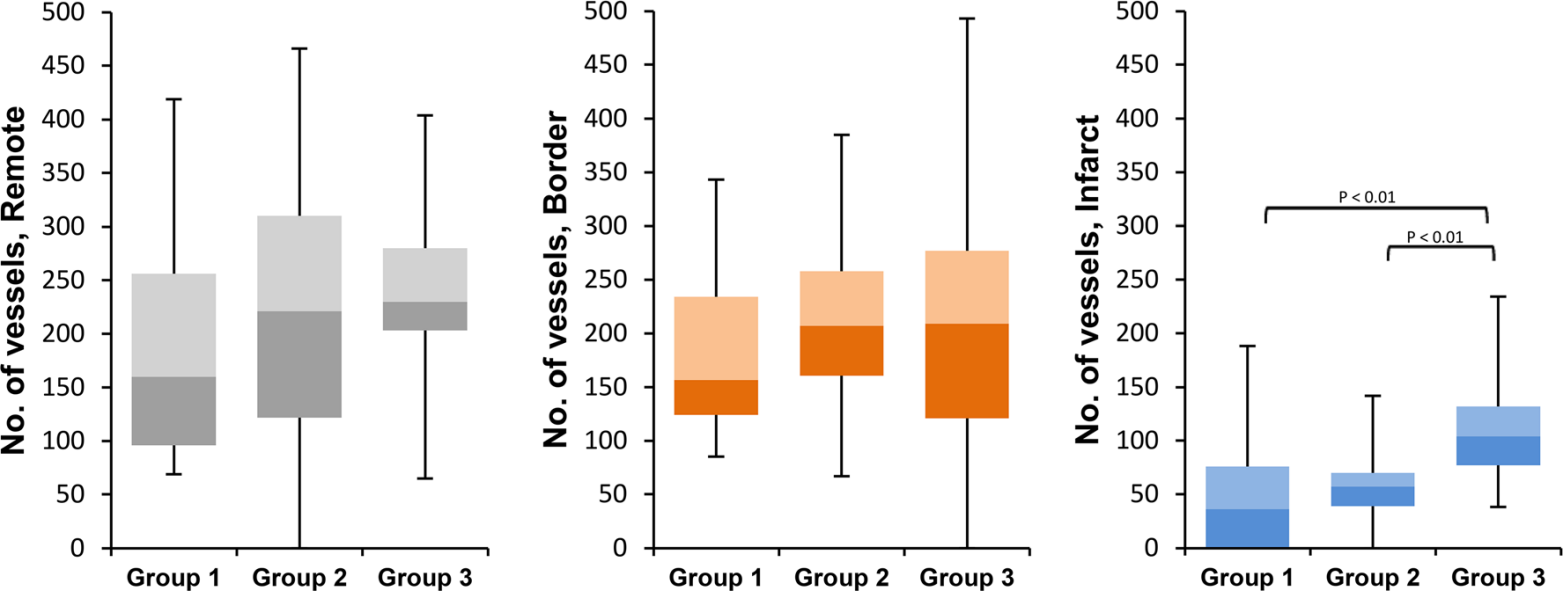
Microcirculatory remodeling in the damaged myocardium after cell delivery 12 weeks post-MI. Microvascular formation determined by Lectin staining at sacrifice was significantly higher in the infarcted zone in group 3 (BMMNC + MSC injection) compared to the other groups (*P* < 0.01). Furthermore, a nonsignificant increase in vessel density was observed in both cell-treated groups in both the remote and border zones (both *P* > 0.1). Group 1: 4 animals, 13 images used for analysis of remote zone, 14 for border zone, 13 for infarct zone. Group 2: 5 animals, 14 images used for analysis of border zone, 23 images for border zone, 18 for infarct zone. Group 3: 6 animals, 23 images used for analysis of remote zone, 27 for border zone, 30 for infarct zone

**Fig. 5:**
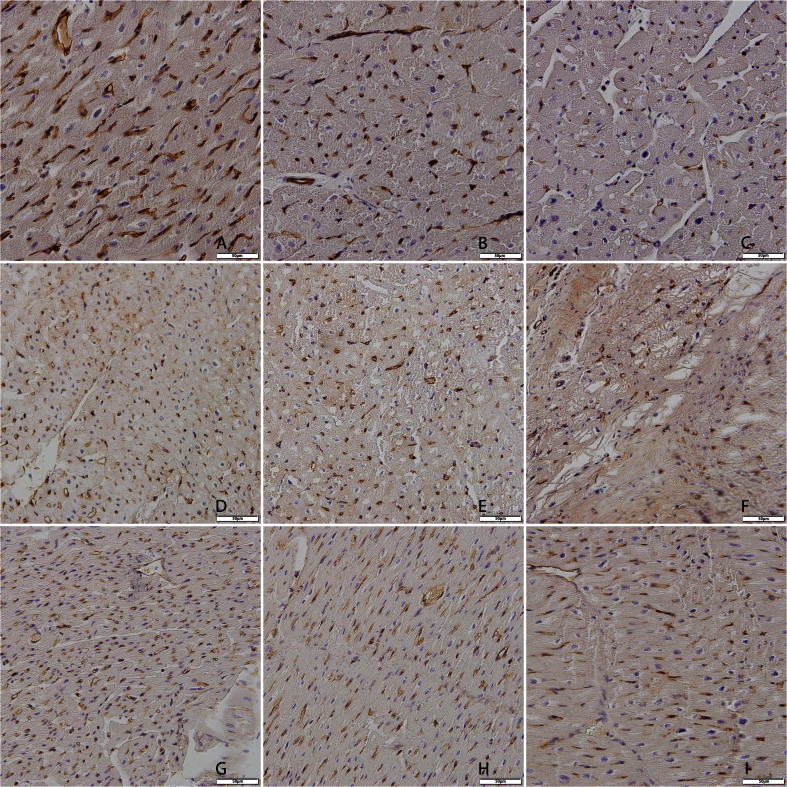
Representative images of Lectin staining at sacrifice from remote area, border zone, and infarcted tissue. **a** Group 1 (placebo + placebo) remote, **b** group 1 border, **c** group 1 infarct. **d** Group 2 (MSC + placebo) remote, **e** group 2 border, **f** group 2 infarct. **g** Group 3 (BMMNC + MSC) remote, **h** group 3 border, **i** group 3 infarct. Magnification ×20

A significantly higher number of capillaries in the infarcted area was seen in group 3 compared to both group 1 and group 2 (median value 104 vs. 36 vs. 57, respectively; *P* < 0.01, Fig. 4). Between groups, no significant difference between the number of vessels in the border zones was found (median value for group 1, 157; group 2, 207; group 3, 209). Also, no difference was found for the remote areas. Within groups, the number of vessels was lower in the infarcted area as compared to border zone and remote area as expected. Furthermore, no significant difference was found in the number of vessels in the remote and border zone. Figure 5 shows representative Lectin-stained images from each group.

Collagen density assessment could be performed on 100 % (225 samples) of the picrosirius red stainings. As expected, infarcted tissue from all groups showed a substantial increase in collagen density compared to tissue from remote areas and border zones.

**Fig. 6:**
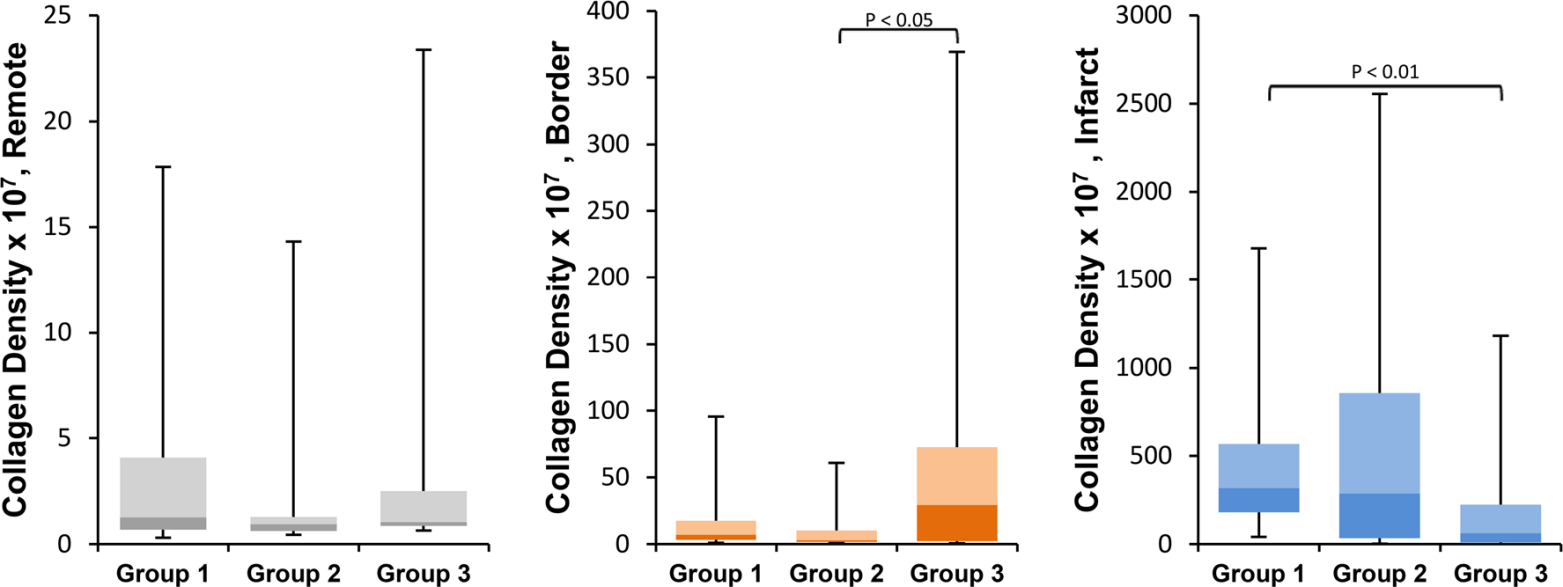
Collagen density after cell therapy. Collagen quantification 12 weeks post infarction of remote areas, border areas, and infarcted areas. A significantly decreased collagen density was observed in the infarcted zone in group 3 (BMMNC + MSC) compared to group 1 (placebo + placebo) (*P* < 0.01). However, in the border zone of group 3, an increase in interstitial fibrosis was measured compared to group 2 (MSC + placebo) (*P <* 0.05). Group 1: 4 animals, 20 images used for analysis per section. Group 2: 5 animals, 25 images used for analysis per section. Group 3: 6 animals, 30 images used for analysis per section. Note differences in *Y*-axis

**Fig. 7:**
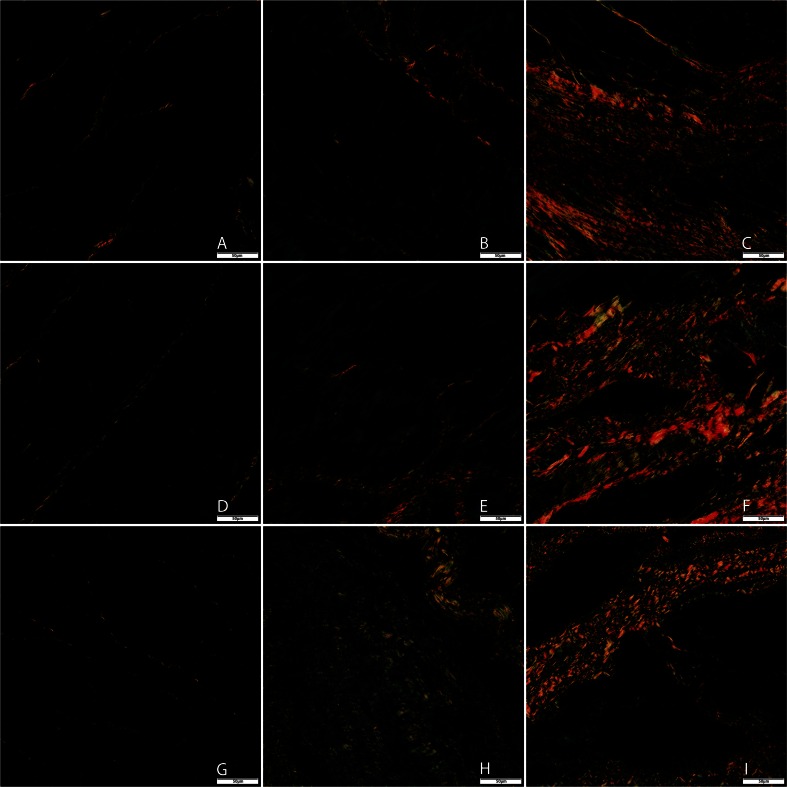
Representative images of picrosirius red staining at sacrifice from remote area, border zone, and infarcted tissue. **a** Group 1 (placebo + placebo) remote, **b** group 1 border, **c** group 1 infarct, **d** group 2 (MSC + placebo) remote, **e** group 2 border, **f** group 2 infarct, **g** group 3 (BMMNC + MSC) remote, **h** group 3 border, **i** group 3 infarct. Magnification ×20

Group 3 showed a significantly lower collagen density in the infarcted area compared to group 1 (median 64 × 10^−7^ vs. 318 × 10^−7^, respectively; *P* < 0.01, Fig. 6), but not group 2 (median 288 × 10^−7^). However, a significantly higher collagen density was observed in the border zone of group 3 compared to group 2 (median 29 × 10^−7^ vs. 3 × 10^−7^, respectively; *P* < 0.05, Fig. 6). Figure 7 shows representative picrosirius red-stained images from each group.

## Discussion

In this study, we performed a comparison between MSC and BMMNC via TE cell delivery in a porcine model of chronic ischemic heart disease. The main novel findings of our study are the following: (1) MSC are superior to BMMNC in improving systolic function, and (2) the delivery strategy of repeated cell injection was safe and feasible. Interestingly, MSC on top of BMMNC led to normalization of LV function, supporting the notion that MSC rather than BMMNC improve systolic function.

### MSC Treatment Improves Systolic Function in Contrast to BMMNC

We performed a head-to-head comparison of treatment with autologous BMMNC and MSC and demonstrated a beneficial effect for MSC on systolic function (EF 11.9 ± 3 %), whereas no effect of BMMNC on LVEF was found compared to placebo (EF −1.6 ± 6 % and −7.8 ± 8 %, respectively).

This was despite the fact that even a slightly higher number of cells were used in the BMMNC group (1.7 × 10^7^ BMMNC vs. 1.0 × 10^7^ MSC). This observation is in line with the results of our large preclinical meta-analysis, showing more benefit of MSC in ischemic heart disease compared to BMMNC [3]. On the contrary, Li et al. did not found significant difference between MSC and BMMNC. However, they infused far more BMMNC than MSC (BMMNC 4.7 ± 1.7 × 10^7^ vs. MSC 6.2 ± 1.6 × 10^5^) [17]. It is known that the number of cells is related to the magnitude of effect [3, 4]. In our study, no statistical difference between injected cell number was observed. Our results may appear to be in contrast with the data from previous clinical studies that did show modest but significant improvements of LVEF after treatment with BMMNC (approximately 3–5 %) [18–20]. However, such studies were mainly performed in the setting of *acute* MI and these effects were predominantly found in subgroups of large infarctions (baseline EF < 48 %) [18]. In fact, several trials with BMMNC in *chronic* patients did not show improvement of systolic function [5, 21, 22]. On the contrary, in a comparable patient cohort, it was demonstrated that indeed MSC were able to improve cardiac function [23]. Recently, a clinical study (TAC-HFT trail) directly compared these cells and demonstrated the safety of both cell types [24]. However, after MSC injection, a reduction in infarct size was observed which was not the case after BMMNC. Unfortunately, this study was not powered to provide a definitive statement on therapeutic efficacy. Taken together, these results provide a robust rationale for a larger trial comparing both cell types to determine whether or not bone marrow stem cells have a clinical future.

### Repeated Cell Injection was Safe but Does Not Further Improve Cardiac Function

Repetitive cell injections led to no serious adverse events (e.g., death, persistent ventricular arrhythmias) but did not further improve systolic function compared to single MSC injection. This is largely due to the lack of an effect of BMMNC; this was surprising and not anticipated. Several studies investigated in particular the effect of repetitive cell transplantations [25]. Our observations are in line with a clinical trial investigating the effect of repeated BMMNC injections in patients with chronic heart failure showing no additional benefit of repeated BMMNC treatment on LVEF. [26] However, Yao et al. demonstrated that repeated BMMNC injection in patients with large acute MI resulted in a significant improvement in ∆LVEF compared to single cell injection [27]. This effect may be explained by the low baseline LVEF values (20–39 %) which were higher in our study. Our results are in line with an observation [28], in which skeletal myoblasts were sequential injected in a chronic infarcted porcine myocardium. Although a different cell type was used, repeated cell injections showed no difference in ∆LVEF (repeated 15.1 % vs. single 11.1 %).

### Histological Effects of MSC Injection

It is postulated that a decreased collagen density and an increase in vascular density in border and/or infarct zone could be the explanation for the observed effects on systolic function in groups 2 and 3. Indeed, collagen density was significantly reduced in the infarcted area in group 3 (BMMNC + MSC) compared to group 1 (placebo + placebo). However, this was not the case for group 2. Surprisingly, the opposite was found for the border zone of group 3. Therefore, the histological analysis of infarcted tissue only partly explains the found effects in the present study.

Second, measurement of capillary density showed a significant increase in the number of microvessels in the infarcted area after BMMNC + MSC injection compared to placebo treatment and MSC + placebo treatment. These data do not directly explain the functional changes although there seems to be a slight trend of increased capillary density in both the border zone and infarcted area of both cell groups; however, a significant increase in the number of vessels was only found in the infarcted area of the BMMNC-primed group as seen in Fig. 4. This suggests a more pronounced angiogenic response after priming with BMMNC before injecting MSC (group 3).

Finally, some MSC were observed in the infarcted tissue by fluorescent microscopy (data not shown), but it is unlikely that the observed effect was caused by differentiation of MSC into cardiac lineages as also suggested by others [29]. However, MSC may lead to prolonged secretion of paracrine factors activating capillary angiogenesis.

### Study Limitations

Our ischemia/reperfusion model resulted in a limited decrease in LVEF (appr. 50 %), but not severe heart failure. This is related to the chosen model (temporary occlusion of LCX for 75 min) and maybe by the fact that animals were treated with similar medication protocols (e.g., beta blockers, which may be cardioprotective) compared to the patients suffering from MI. Nevertheless, significant effects on LVEF were observed.

The porcine model is considered the best possible model to resemble the clinical situation, although major differences exist (e.g., risk factors, cell isolation protocols [30], comorbidity, follow-up duration), which prevent direct extrapolation to patient management. Nevertheless, our group demonstrated that large animal models can accurately predict human clinical outcome and these models are frequently used for translational purposes [3].

Care was taken to perform adequate analysis of collagen and vascular density; however, both sampling error and staining issues may have had impact on this histological assessment. By analysing five sections per sample, we attempted to minimize this bias.

Nowadays, cardiac MRI is considered the gold standard to measure LVEF and volumes. However, due to practical reasons, we performed echocardiographic and pressure-volume loop analysis. These techniques are still considered reliable, reproducible, and a valid measure of LV function and are therefore most often used in preclinical research models.

Finally, our results show that the anticipated synergistic effect by pretreatment with BMMNC before MSC treatment in group 3 was suggested by the increased capillary density, but was not represented in functional endpoints. Knowing this, a fourth group comparing repetitive MSC injections would have been helpful to elucidate this effect for repetitive MSC treatment. Nevertheless, we feel that the take-home message from this particular group is now reassuring that indeed MSC outperform BMMNC in this setting.

## Conclusions

We demonstrated that MSC are more potent in terms of improvement of LVEF than BMMNC in a chronic model for ischemic heart disease. Our data do not support strategies using repetitive injections, although using different combinations of cells may be of value in more severe heart failure. These data should encourage researchers and clinicians to focus future studies on other cell types (i.e., MSC) than BMMNC.
